# Selenium and Sulphur as Elements Modifying Plant Quality: Assessment of the Content of Organic and Mineral Nitrogen Forms in Wheat

**DOI:** 10.3390/molecules30234541

**Published:** 2025-11-25

**Authors:** Marzena S. Brodowska, Magdalena Kurzyna-Szklarek, Mirosław Wyszkowski

**Affiliations:** 1 Department of Agricultural and Environmental Chemistry, University of Life Sciences in Lublin, Akademicka 15 Str., 20-950 Lublin, Poland; 2 Department of Agricultural and Environmental Chemistry, University of Warmia and Mazury in Olsztyn, Łódzki 4 Sq., 10-727 Olsztyn, Poland

**Keywords:** sulphur, selenium, spelt wheat, common wheat, N forms

## Abstract

For many years, deficiencies in selenium (Se) and, sometimes, sulphur (S) have been observed in populations in most European countries and other parts of the world. This study aimed to determine the effect of selenium (0, 10 and 20 g Se ha^−1^) and the date of its application (in the tillering phase—BBCH 22–24 and in the stem elongation phase—BBCH 31–34) and sulphur application (0, 15 and 30 kg S ha^−1^) on the content of various forms of nitrogen (total nitrogen, protein nitrogen, ammonium nitrogen, and nitrate nitrogen) in winter spelt wheat and winter common wheat. Selenium application influenced nitrogen distribution in wheat. In spelt, grain total-N decreased by 4%, while common wheat straw increased by 13%. Application timing had no significant effect on total-N. Sulphur slightly raised total-N in common wheat grain and straw. Selenium reduced protein-N in spelt grain by 7% but increased it in common wheat straw by 18%. Ammonium-N in straw rose by 24–27%, while nitrate-N declined in grain and straw of both species. Sulphur-enhanced mineral N forms (ammonium-N by 6–16%, nitrate-N by 7–24%), especially in the early stages. Se–S fertilization strategies should consider their competitive uptake and impact on N metabolism.

## 1. Introduction

Obtaining high yields of good quality requires providing plants with adequate amounts of nutrients that fully cover their nutritional needs [1,2]. Selenium (Se) deficiencies have been observed in the populations of most European countries and other places of the world for many years. The key factor contributing to the deficiency of this element is the reduction in its emissions from anthropogenic sources and the gradual depletion of natural selenium resources [3].

It is assumed that higher plants may have lost the necessary Se metabolism during their evolution [4]. Although it is not considered essential for higher plants, it is considered a beneficial element, stimulating their growth even in small amounts [5]. The mechanisms of this beneficial effect are still largely unknown but may be related to increased antioxidant activity [6]. Unfortunately, elevated Se levels are toxic to most plants due to the nonspecific incorporation of Se into sulphur (S) compounds and oxidative stress [7].

Organisms that consume only plant-based diets may develop diseases caused by selenium deficiency. This is due to the varying distribution of this element in the soil and insufficient uptake by plants. Therefore, management strategies aimed at minimizing Se deficiencies and related disorders should focus on the relationship between agricultural food products and the content and availability of Se in the soil [8]. Methods of supplementing selenium deficiency in the diet mainly include supplementation and fortification, i.e., fortification of livestock feed and plants. One of the most promising approaches to combating low Se transfer from soil to the food chain is agronomic Se biofortification, which is defined as increasing the concentration of bioavailable essential elements in the edible parts of crops [9]. Increased intake of this micronutrient, as well as its bioavailability and accumulation by edible plants, can be achieved by adapting cropping systems, selecting Se-accumulating varieties, and using modern genetic engineering technologies [10].

Plants can absorb Se from the soil in the form of selenate (VI) (SeO_4_^2−^), selenite (IV) (SeO_3_^2−^), and organic compounds [11]. The former are present in the soil and are absorbed by the roots, while the leaves absorb organic forms present in the air [12]. Plants do not absorb elemental Se and metal selenides. Water-soluble forms of Se are easily absorbed, but this process depends on the species and variety of the plant [13,14].

Plants that accumulate selenium during uptake clearly favour this element and can accumulate up to several mg kg^−1^ of dry weight, while those that do not accumulate Se rarely contain 0.1 mg kg^−1^ of dry weight [15]. The current view is that Se is not an essential element for plants [6], which is why plants have not developed specific uptake pathways for it. Since Se is a chemical analogue of sulphur, the uptake of selenates (VI) follows the same pathway as sulphates (VI). Plants use sulphur transporters, which have a high affinity for Se, for membrane transport [16]. Under conditions of S deficiency, the expression of genes responsible for encoding sulphate permeases and enzymes responsible for Se/S metabolism increases, resulting in increased uptake of selenates (VI) [17].

The main factor determining the bioavailability of Se in food sources enriched with this element is its speciation. Se can occur in both inorganic and organic forms, but the predominant form of Se extracted from plants by enzymatic hydrolysis is organic—selenomethionine (SeMet), methylselenocysteine (MeSeCys), γ-glutamyl-methyl-selenocysteine (γ-Glu-MeSeCys) [18,19].

Sulphur is an essential element for the proper growth and life of plants and animals [20]. The S requirement of higher plants depends on the plant species as well as the yield. Plant sulphur requirements can be defined as the minimum amount of S that is sufficient for a plant to obtain the maximum yield of the desired quality [21]. Quantitatively, this requirement is similar to or even exceeds the requirement for phosphorus. *Brassica* plants take up more sulphur than phosphorus. The sulphur requirement also varies depending on the growth and development stage of the plant within a given species [22].

The necessity of sulphur stems mainly from the fact that in plant organisms, this element is a component of amino acids, bitter oils, and other organic compounds and also accumulates in tissues in mineral form as sulphate. It participates in numerous metabolic and physiological processes in plants that are of great biological, protective, and economic importance [23,24]. Sulphur compounds are crucial in many cellular processes, including oxidation and reduction reactions, detoxification of heavy metals and xenobiotics, carbohydrate and fat formation processes, and photosynthesis, as well as chlorophyll and lignin synthesis [25,26]. Sulphur is a component of amino acids, peptides and proteins, enzymes, and vitamins. In addition, it increases plant resistance to stress conditions by participating in the plant’s defense system against pests and diseases [27].

It is estimated that 90% of the total S content in plants consists of sulphur amino acids, including cysteine and methionine, which determine the content and biological value of protein. Cysteine easily releases hydrogen from the thiol group (-SH), and when two cysteine molecules release hydrogen, one cystine molecule is formed. During this process, a disulfide bridge is formed, which is very important in maintaining the spatial structure of the protein. Methionine, on the other hand, easily donates methyl groups. Both of these amino acids are precursors of glutathione (GSH), thiamine, biotin, coenzyme A, lipoic acid, thioredoxin, and sulfolipids [28,29]. In addition, sulphur is a component of glucosinolates, which are characteristic of plants of the *Brassicaceae* (*Cruciferae*) family, and allin, which occurs mainly in plants of the *Allium* genus. Both glucosinolates and auxins perform defensive functions in plants, and glucosinolates can additionally store sulphur during periods of low supply [30,31,32].

Selenium metabolism in plants is linked to the sulphur assimilation pathway, as selenates use the same transporters and enzymes as sulphates [33]. The reduction produces sulphur amino acid analogues such as selenocysteine and selenomethionine [34]. These compounds influence protein synthesis and thus nitrogen metabolism, which is crucial for plant growth [35]. High sulphur availability promotes the biosynthesis of sulphur amino acids, while its deficiency increases selenium uptake, which alters the balance in N metabolism [33]. Selenium in moderate doses can stimulate amino acid synthesis and antioxidant enzyme activity, but its excess disrupts nitrogen assimilation [35]. Studies have shown that simultaneous Se and S fertilization modulates amino acid and sugar levels, affecting the integration of S–Se–N pathways [34]. In addition, selenium nanoparticles regulate nitrogen metabolism, including the biosynthesis of arginine and glycine–serine [36].

In the case of selenium and sulphur deficiencies, biofortification of plants with these elements seems to be necessary [37]. For this reason, studies were conducted to determine the effect of selenium and sulphur on the content of various forms of nitrogen (total nitrogen, protein nitrogen, ammonium nitrogen, nitrate nitrogen) in winter spelt wheat and winter common wheat. A research hypothesis was put forward, assuming that varying doses of selenium and the date of its application, as well as the doses of sulphur, have a positive effect on the content of nitrogen forms in the grain and straw of spelt wheat and common wheat.

## 2. Results

### 2.1. Total Nitrogen of Wheat

The average total nitrogen content in spelt wheat grain was 20.53 g kg^−1^ dry matter, and in common wheat, it was 20.04 g kg^−1^ dry matter (Table 1). Fertilization with selenium in both doses resulted in a significant reduction in the total nitrogen content in spelt wheat grain (by only 4%) but had no significant effect on the total nitrogen content in common wheat grain. However, different timing of selenium application to the soil did not result in a targeted change in the total nitrogen content in spelt wheat and common wheat grains. Sulphur fertilization did not cause any clear changes in the total nitrogen content in spelt wheat grain and in common wheat grain.

In spelt wheat straw (Table 2), the application of the above-mentioned components did not result in statistically significant changes in total nitrogen content. Selenium fertilization resulted in an increase in total nitrogen content (by 13%) in the straw of common wheat, while the timing of selenium application did not cause any changes in the total nitrogen content in spelt wheat and common wheat. The total nitrogen content in common wheat straw increased (by 4%) as a result of fertilization with a lower dose of sulphur.

### 2.2. Protein Nitrogen of Wheat

Selenium fertilization was associated with a decrease in the content of protein nitrogen in spelt wheat grain (Table 3). Fertilization with a lower dose of selenium resulted in a 4% decrease, and a higher dose resulted in a 7% decrease in spelt wheat grain. Fertilization with both lower and higher doses of selenium slightly reduced the protein nitrogen content in common wheat grain. Later application of selenium was associated with slightly lower protein nitrogen content in spelt wheat grain. On the other hand, higher protein nitrogen content (by 5% on average) in common wheat grain was observed in the case of later selenium application, regardless of the degree of sulphur fertilization. Sulphur fertilization of spelt wheat did not result in a clearly directed change in protein nitrogen content, but the application of a higher dose reduced the protein nitrogen content in common wheat grain by 6% compared to the control objects.

Selenium fertilization, as well as the timing of its application, did not result in a clear change in the protein nitrogen content of spelt wheat straw (Table 4). The application of a lower dose of selenium to the soil environment resulted in an increase in the protein nitrogen content in common wheat straw by 10%, and a higher dose by as much as 18%. The second date of selenium application was associated with slightly higher protein nitrogen contents in common wheat straw. Sulphur fertilization resulted in a decrease in protein nitrogen content in spelt wheat straw by 7% and 3%, respectively.

### 2.3. Ammonium Nitrogen of Wheat

The ammonium nitrogen content in spelt wheat grain decreased in most experimental sites with the addition of selenium to the soil, with the application of a lower dose resulting in a change of as much as 19% (Table 5). The application of selenium did not cause any clear changes in the ammonium nitrogen content in common wheat grain. The application of selenium at the second date was associated with a 7% decrease in ammonium nitrogen content in spelt wheat grain and a 6% decrease in common wheat grain, compared to previously fertilized plots. Fertilization with sulphur in both doses contributed to an increase in the ammonium nitrogen content in spelt wheat grain by 7% and 13%, respectively, compared to objects without sulphur. The addition of a lower dose of sulphur to the growth environment of common wheat resulted in a 10% higher ammonium nitrogen content in its grain. The application of a higher dose did not cause any significant changes in the ammonium nitrogen content compared to the control plots without sulphur.

Selenium fertilization increased the ammonium nitrogen content in spelt wheat straw by 22% and 27%, respectively (Table 6). Common wheat straw showed an increase in ammonium nitrogen content after the addition of selenium to fertilizers (by 13% and 24%, respectively). Later application of selenium resulted in a 6% lower ammonium nitrogen content in spelt wheat straw compared to previously fertilized objects. However, the timing of selenium application did not have a clear effect on the ammonium nitrogen content in common wheat straw. In most cases, sulphur-treated plots had higher ammonium nitrogen content in spelt wheat straw compared to the same combinations without sulphur application. The application of a lower dose of sulphur resulted in a 6% increase in the ammonium nitrogen content in the straw of this plant. Fertilization with sulphur, regardless of the dose, resulted in an increase in the ammonium nitrogen content in common wheat straw. The application of a lower dose resulted in an increase of 16%, and a higher dose resulted in an increase of 5%.

### 2.4. Nitrate Nitrogen of Wheat

Selenium fertilization in most experimental facilities significantly reduced the nitrate nitrogen content in spelt wheat and common wheat grains (Table 7). The application of a lower dose was associated with a 9% and 5% reduction in nitrate nitrogen content in spelt wheat and common wheat grains, respectively, while a higher dose resulted in a 24% reduction in spelt wheat grains. Subsequent selenium fertilization did not promote nitrate nitrogen accumulation, as a decrease in its content was observed in both spelt wheat and common wheat grains. Later application of selenium was also associated with a 9% reduction in nitrate nitrogen content in common wheat grain compared to plants fertilized earlier. Fertilization with both doses of sulphur caused a significant increase in nitrate nitrogen content (by 8% and 4%, respectively) in spelt wheat grain, while fertilization with a lower dose of sulphur caused it in common wheat grain (by 24%).

The addition of selenium to fertilizers in most facilities resulted in a reduction in nitrate nitrogen content in spelt wheat straw (by 17% and 15%, respectively) and in common wheat straw (by 17% and 18%, respectively), compared to facilities without selenium (Table 8). The timing of selenium application was not associated with a clear change in the nitrate nitrogen content of spelt wheat straw. However, later selenium application resulted in a 22% lower nitrate nitrogen content in common wheat straw. Sulphur fertilization was associated with an increase in nitrate nitrogen content in spelt wheat straw, even by 7% after application of a higher dose of this component. In common wheat straw, however, fertilization with a higher dose of sulphur resulted in a 15% reduction in nitrate nitrogen content.

### 2.5. Share of Protein Nitrogen in Total Nitrogen of Wheat

In order to assess protein synthesis efficiency, the influence of the experimental factors used on the percentage share of the nitrogen fraction in its total content was analyzed (Table 9 and Table 10). In both test plants, this indicator took on similar values. In our own studies, no average percentage of protein nitrogen below 75% was recorded, which indicates a very good supply of sulphur to the test plants.

The presence of selenium in the fertilizer dose, as well as the dose and timing of its application, were also not associated with clearly directed changes in this index in spelt wheat grain and common wheat grain. In spelt wheat grain, no significant changes in the percentage of protein nitrogen in the total nitrogen content were observed under the influence of sulphur fertilization. However, fertilization with a higher dose of sulphur resulted in an increase in the percentage of protein nitrogen in the total nitrogen content by 5% in common wheat grain.

As in the case of test plant grains in straw, no significant changes in the percentage of protein nitrogen in total nitrogen content were observed in either spelt wheat or common wheat under the influence of the used elements.

## 3. Discussion

According to Argento et al. [38] and Yu et al. [39], the average total nitrogen content in wheat grain is approximately 19.5 g kg^−1^ dry matter. The results obtained in our own research achieved similar values. These data are also consistent with the results of other authors concerning similar total nitrogen content in plants [40].

In the experiments conducted by Wang et al. [41] and Schiavon et al. [35], selenium fertilization was associated with a decrease in nitrogen content in wheat, while in the studies by Ducsay et al. [42] and Zhang et al. [43], it did not result in significant changes in nitrogen content in common wheat grain. This may be related to the effect of selenium on nitrogen metabolism, including the activity of enzymes such as nitrate reductase and glutamine synthetase, which are key to nitrogen assimilation in plants. Selenium may compete with sulphur in metabolic pathways, leading to disturbances in the synthesis of nitrogen-containing amino acids. Relatively small changes in nitrogen content in wheat grain may result from the greater tolerance of cereals to the presence of selenium and from differences in the mechanisms of transport and accumulation of this element in generative tissues. Wheat may be able to compensate for selenium stress by redistributing nitrogen from vegetative to generative organs. Research indicates that, under conditions of stress or limited nutrient availability, wheat intensifies the mobilization of nitrogen from its leaves and stems to its ears in order to maintain grain development [44]. This mechanism is crucial when selenium stress affects nitrogen metabolism, as the plant compensates for deficiencies by increasing the redistribution of nitrogen to generative organs [43]. In studies by Tabak et al. [45], sulphur fertilization resulted in an increase in nitrogen content in winter wheat grain. This effect can be explained by the synergistic action of sulphur and nitrogen in protein biosynthesis processes. Sulphur is essential for the synthesis of amino acids such as cysteine and methionine, which are precursors of many nitrogen compounds. Its presence promotes more efficient nitrogen utilization by plants, which translates into higher nitrogen accumulation in the grain. Similarly, in the experiment by Liu et al. [46], sulphur fertilization resulted in an increase in the total nitrogen content in spring wheat straw. The increase in nitrogen content in straw may be the result of improved overall plant nutrition, leading to increased synthesis of nitrogen compounds in vegetative tissues [47]. In addition, sulphur may influence the expression of genes related to nitrogen transport and storage, which promotes its accumulation in straw [48]. It is worth noting that the effects of fertilization with micronutrients such as selenium and sulphur are highly dependent on the plant species, developmental stage, chemical form of the element, and environmental conditions [49,50]. Therefore, further research should focus on understanding the molecular mechanisms of interaction between these elements and nitrogen, which may contribute to the optimization of fertilization strategies in precision agriculture.

The results of the study indicate that selenium fertilization was not associated with a clear change in the protein nitrogen content of spelt wheat straw. The lack of clear effects may result from the specificity of this species, which is characterized by a different nitrogen and sulphur metabolism compared to common wheat. Spelt wheat, as a more primitive form, may be less sensitive to micronutrients applied in the form of fertilizers, which translates into a limited response in terms of protein synthesis [41,51].

The timing of selenium application also did not cause significant changes in the protein nitrogen content of spelt wheat straw in our study, which may suggest that for this species, the physiological window for effective selenium utilization is narrower or shifted compared to common wheat. In contrast, the application of a lower dose of selenium to the soil environment increased the protein nitrogen content in common wheat straw by 10%, and a higher dose by as much as 18%. This result confirms that selenium can act as a stimulator of nitrogen metabolism, probably by influencing the activity of enzymes involved in amino acid and protein synthesis [43]. The second application date of selenium was associated with slightly higher protein nitrogen content in common wheat straw, which may be the result of a better match to the phase of intensive vegetative growth, when plants have an increased demand for micronutrients supporting protein synthesis [51,52].

In turn, in our study, sulphur fertilization resulted in a decrease in the protein nitrogen content in spelt wheat straw, which may be surprising given the synergistic effect of sulphur and nitrogen in protein synthesis. A possible explanation is antagonism between sulphur and other nutrients or disturbances in metabolic balance, especially if sulphur doses were too high or applied at a suboptimal time [49,53]. In studies by other authors [49,53,54], information on the opposite relationship can be found, as most experiments reported an increase in protein nitrogen content following sulphur fertilization. This is related to the fact that adequate sulphur supply to plants in the early stages of growth results in increased protein accumulation [49,50,53].

These results indicate the need for further research on the interactions between micronutrients and major nutrients in different cereal species. Taking into account the specific characteristics of the genotype, the stage of development, and the chemical form of the fertilizers used may allow for a better understanding of the mechanisms regulating protein accumulation in plants [55].

The results of our study indicate that selenium fertilization in most experimental sites led to a decrease in the ammonium nitrogen content in spelt wheat grain. This effect may be related to the influence of selenium on nitrogen metabolism in plants, particularly on the activity of enzymes such as glutamine synthetase and nitrate reductase, which regulate the conversion of mineral forms of nitrogen into organic compounds. Selenium may limit the accumulation of ammonium forms by stimulating their faster incorporation into amino acid synthesis pathways [43]. In turn, sulphur fertilization, regardless of the dose, contributed to an increase in the ammonium nitrogen content in spelt wheat grain, and a lower dose of sulphur also increased its content in common wheat grain. Sulphur, as an element essential for the synthesis of sulphur amino acids, may promote more efficient utilization of ammonium forms in protein biosynthesis, which explains their higher levels in generative tissues [49]. The effect of sulphur fertilization was more pronounced—in most cases, it led to an increase in the ammonium nitrogen content in the straw of both species. This may be due to an improvement in the overall nutritional status of the plants and the effect of sulphur on the expression of genes related to nitrogen transport and storage [48]. It is worth noting that the effects of fertilization with micronutrients such as selenium and sulphur are strongly dependent on the plant species, developmental stage, chemical form of the element, and environmental conditions [50].

The results of our study indicate that selenium fertilization significantly reduced the nitrate nitrogen content in spelt wheat and common wheat grains. This phenomenon can be explained by the multifaceted effect of selenium on plant physiology, especially in the context of nitrogen metabolism. Selenium, as a microelement with antioxidant properties, can modulate the activity of enzymes involved in nitrogen metabolism, such as nitrate reductase, thereby limiting the excessive accumulation of mineral forms, including nitrates [43]. From the point of view of food quality, reducing the nitrate content in grain is beneficial. Nitrates (V) can be reduced to nitrites (III) in the digestive tract. Nitrates (V) can be reduced to nitrites (III) in the digestive tract of humans and animals, which is associated with the risk of nitrosamine formation—compounds with potential carcinogenic effects [56].

Contrary to the results of our own research, many authors [25,49] have reported a positive effect of sulphur on reducing the accumulation of mineral forms of nitrogen. In contrast, Ghafoor et al. [47] and Hřivna et al. [57] report an increase in the nitrate form of nitrogen in wheat grain under the influence of sulphur fertilization. Other authors [25,49,50,53] also emphasize the beneficial effect of sulphur on nitrogen metabolism and the reduction in its mineral forms. This fact is explained by the fact that in plants with sulphur deficiency, the proportion of non-protein forms of nitrogen increases, among other things, due to the lack of essential amino acids, including cysteine and methionine. The accumulation of nitrate nitrogen may be associated with a decrease in the biological value of plants, as it can have harmful effects. This is due to the fact that nitrates (V) can be reduced to nitrites (III) in the digestive tract of animals and humans [56].

According to Gondek [58], a protein fraction content of 75% and above is the value that determines the optimal supply of sulphur to plants. In wheat exposed to sulphur deficiency, this index may be lower than 25%. The lack of significant changes in the percentage of protein nitrogen in the total nitrogen content in spelt and common wheat straw, despite the use of different fertilizer components in our study, may indicate the stability of the mechanisms regulating protein synthesis in the vegetative tissues of these species. Straw, as a by-product with a lower metabolic priority than grain, may be less sensitive to changes in the availability of micro- and macronutrients [46]. In the case of selenium, its effect on nitrogen metabolism may be more evident in generative organs, where intensified protein synthesis is crucial for crop quality. In vegetative tissues such as straw, selenium may not have such a strong stimulating effect, which explains the lack of significant changes in the proportion of protein nitrogen. Additionally, selenium may influence the redistribution of nitrogen in the plant, preferentially directing it to the grain, which limits its accumulation in straw [43]. Similarly, sulphur, although it plays a key role in the synthesis of sulphur amino acids (cysteine, methionine), may not significantly affect the proportion of protein nitrogen in straw if its action is focused on improving the quality of proteins in the grain. High sulphur availability may promote more efficient nitrogen use in protein synthesis but does not necessarily lead to changes in proportions in tissues of lower productive importance [49]. It is also worth noting that the proportion of protein nitrogen in total nitrogen can be stabilized by plant homeostatic mechanisms that maintain a balance between nitrogen forms regardless of fertilization conditions. Such stability may be beneficial from the point of view of plant nitrogen management, especially under conditions of variable nutrient supply [59].

Selenium and sulphur are chemically similar, meaning plants primarily absorb selenium in the form of selenates via the same transporters that are responsible for absorbing sulphates. This leads to competition at the root level [33]. High sulphur availability limits selenium accumulation, whereas deficiency increases selenium uptake by intensifying sulphate transporter expression [34]. Once inside cells, selenium enters the sulphur assimilation pathway via enzymes such as ATP-sulfurylase and APS-reductase. This results in the formation of analogues of sulphur amino acids, namely selenocysteine and selenomethionine [35]. These compounds influence protein synthesis and, consequently, nitrogen metabolism—both of which are essential for plant growth [33].

Moderate selenium doses can stimulate amino acid synthesis and antioxidant enzyme activity, but excessive Se interferes with nitrogen assimilation by reducing nitrate reductase (NR) and glutamine synthetase activity, as well as affecting molybdenum availability, which is an NR cofactor [36,60]. Excessive selenium concentrations also cause oxidative stress, leading to protein degradation and reduced accumulation in tissues [61]. Consequently, despite the fact that low doses of selenium can enhance N metabolism, plants exhibit lower total and protein nitrogen content [62].

On the other hand, sulphur supports the accumulation of mineral nitrogen by increasing the efficiency with which it is taken up and assimilated. Availability of S stimulates nitrate reductase activity and the synthesis of sulphur amino acids, improving nitrogen incorporation into proteins [63,64]. Under conditions of sulphur deficiency, plants accumulate nitrogen in the form of nitrates and amides because the processes of reduction and incorporation into proteins are limited [65]. Therefore, while sulphur itself does not inhibit nitrogen assimilation, a lack of sulphur can cause disturbances to this process. An adequate supply of sulphur improves nitrogen uptake and its effective use in protein synthesis.

Studies have shown that simultaneous fertilization with selenium and sulphur modulates the levels of amino acids and sugars, thereby influencing the integration of S–Se–N pathways [34]. Furthermore, selenium nanoparticles can regulate nitrogen metabolism, including the biosynthesis of arginine and glycine–serine, opening up new avenues for plant biofortification [36]. Properly balancing the supply of selenium (Se) and sulphur (S) is crucial for crop quality and avoiding metabolic disorders.

## 4. Materials and Methods

### 4.1. Methodological Assumptions

The research was conducted based on a three-year (2015–2018) field experiment carried out at the Experimental Station of the University of Life Sciences in Lublin, located in Czesławice, Poland (51°18′23″ N, 22°16′02″ E). The experiment was established on loamy soil [66], formed from loess, characterized by pH_KCl_—6.3, mineral nitrogen (0.83 mg NH_4_-N kg^−1^ d.m. and 22.39 mg NO_3_-N kg^−1^ d.m.), high phosphorus (105.5 mg P kg^−1^ d.m.) and magnesium (59.0 mg kg^−1^ d.m.) content, and medium sulphur (10.4 mg SO_4_ kg^−1^ d.m.) and potassium 134.5 mg K kg^−1^ d.m.) content. Two test plant species were used in the study: winter spelt wheat (*Triticum spelta* L., Rokosz variety, Hodowla Roślin Strzelce Sp. z o.o., Strzelce, Poland) and winter common wheat (*Triticum aestivum* L., Astoria variety, Poznańska Hodowla Roślin, Tulce, Poland).

The experiment had three factors. The first factor was three levels of sulphur fertilization (S_0_—0 kg S ha^−1^, S_1_—15 kg S ha^−1^, S_2_—30 kg S ha^−1^), the second factor was three levels of selenium fertilization (Se_0_—0 g Se ha^−1^, Se_1_—10 g Se ha^−1^, Se_2_—20 g Se ha^−1^), and the third factor was two dates of selenium application: in the tillering phase (BBCH 22–24) and in the stem elongation phase (BBCH 31–34). S and Se were applied at three levels, while the application timing was applied at two levels. The plants were sown in the first ten days of October and harvested in the second ten days of August. Sulphur was applied before sowing in the form of ammonium sulphate ((NH_4_)_2_SO_4_), while selenium was applied in the form of sodium selenite (IV) (Na_2_SeO_3_). Nitrogen was applied before sowing in the form of ammonium nitrate (NH_4_NO_3_) at a dose of 20 kg N ha^−1^, and then supplemented in spring to a total dose of 80 kg N ha^−1^: 40 kg N ha^−1^ in the tillering phase (BBCH 22–24), taking into account nitrogen from ammonium sulphate, and 20 kg N ha^−1^ in the stem elongation stage (BBCH 31–34). Phosphorus and potassium were applied before sowing at doses of 60 kg P_2_O_5_ ha^−1^ and 80 kg K_2_O ha^−1^, respectively. Selenium was delivered to the plants foliarly by spraying the plants with an aqueous solution containing this element. Four fungicide treatments were carried out against fungal diseases. Yamato 303 SE (methyl thiophanate, 233 g L^−1^ + tetraconazole, 70 g L^−1^) at a dose of 1.5 L ha^−1^ in the BBCH 30–31 phase; Optan 183 SE (pyraclostrobin, 133 g L^−1^ + epoxiconazole, 50 g L^−1^) at a dose of 1.5 L ha^−1^ in the BBCH 30–59 phase; Wirtuoz 520 EC (prochloraz, 320 g L^−1^ + tebuconazole, 160 g L^−1^ + proquinazid, 40 g L^−1^) at a dose of 1 L ha^−1^ in the BBCH 20–59 phase; Tilt Turbo 575 EC (propiconazole, 125 g L^−1^ + fenpropidin, 450 g L^−1^) at a dose of 0.9 L ha^−1^ in the BBCH 30–59 phase. Herbicide treatment was carried out in the BBCH 21–29 stage of wheat with Chisel 75 WG (thifensulfuron-methyl, 682 g kg^−1^ + chlorosulfuron, 68 g kg^−1^) at a dose of 60 g ha^−1^ with the addition of a 0.1% solution of the Trend 90 EC adjuvant (ethoxylated isodecyl alcohol, 900 g L^−1^). Wheat was protected against pests by applying Decis Mega 50 EW (deltamethrin, 50 g L^−1^) at a dose of 0.2 L ha^−1^.

Spring wheat and common wheat were harvested in a single stage at the stage of full physiological maturity. After harvesting, representative samples of plant material (grain and straw) were taken for further laboratory analysis in accordance with the applicable research procedures.

### 4.2. Laboratory and Statistical Analysis Methods

During harvesting, plant material intended for laboratory analysis was collected from the fragment of each experimental plot (area 0.25 m^2^). After separating the grain and straw using a threshing machine, the plant material was dried at 60 °C until a constant weight was obtained and then ground in an electric grinder. The resulting homogenate was used as a substrate for determining the content of various forms of nitrogen. The ammonium nitrogen (NH_4_^+^-N) content was determined colorimetrically using the Nessler method in trichloroacetic acid (CCl_3_COOH) extract, while nitrate nitrogen (NO_3_^−^-N) was determined colorimetrically using sodium salicylate [67]. The protein nitrogen content was determined by distillation after prior extraction with trichloroacetic acid [68]. In addition, grain and straw samples were mineralized in concentrated sulphuric acid (H_2_SO_4_) with the addition of a catalyst (hydrogen peroxide) according to the Kjeldahl procedure. The total nitrogen content in the resulting solution was determined by distillation [69,70].

The data obtained during the study were subjected to statistical analysis using three-factor analysis of variance (ANOVA), performed using the Statistica package [71,72]. In order to identify significant differences between the means, Tukey’s post hoc test (HSD) was used at a significance level of *p* = 0.05 [62]. Based on the test, the least significant differences (LSD) and statistically homogeneous groups of objects were determined [71]. The values presented in the tables for the interactions of factors A × B × C marked with the same letters do not differ significantly statistically, which confirms their belonging to the same homogeneous group.

## 5. Conclusions

The application of selenium was associated with a significant decrease in total nitrogen content in the grain of spelt wheat by 4% (unlike in the grain of common wheat) and a significant increase in total nitrogen content by 13% in the straw of common wheat. The timing of selenium application did not clearly differentiate total nitrogen content in the grain and straw of the test plants. Sulphur fertilization insignificantly increased the total nitrogen content in the grain and straw of common wheat. The application of selenium resulted in a decrease (by 7%) in the protein nitrogen content in the grain of spelt wheat and an increase (by 18%) in the common wheat straw. The presence of sulphur in the soil environment did not clearly differentiate the protein nitrogen content in the grain and straw of the test plants. The presence of selenium in the soil resulted in an increase in ammonium nitrogen content in the straw of both plants (by 27% and 24%) and a decrease in nitrate nitrogen content in the grain of spelt wheat (by 9% and 24%) and straw (by 17% and 18%) of the tested plants. Sulphur fertilization (especially the first dose) promoted the accumulation of mineral forms of nitrogen (ammonium-N by 6–16%, nitrate-N by 7–24%) in both the grain and straw of spelt and common wheat. However, these values were not higher than those most commonly found in these plants.

Strategies for the use of selenium–sulphur fertilizers should take into account both the competition of Se and S in uptake and their impact on nitrogen metabolism. In soils low in sulphur, it is advisable to combine selenium with sulphur because the presence of S improves the efficiency of nitrogen assimilation and reduces the risk of excessive Se accumulation in tissues. Balanced Se–S fertilization allows you to maintain the proper synthesis of sulphur amino acids and proteins, which is crucial for the quality of the crop and food safety. Research in this area is of great importance for agricultural practices because it allows the development of selenium biofortification strategies without a negative impact on nitrogen metabolism and plant growth.

## Figures and Tables

**Table 1:** Total nitrogen content (g kg^−1^ d.m.) of grain of spelt wheat (*Triticum spelta* L.) and common wheat (*Triticum aestivum* L.).

Selenium Dose (B)	Sulphur Dose (A)			Average
	S0	SI	SII	
Spelt wheat (*Triticum spelta* L.)				
1st selenium application date (C)				
Se0	20.94 ^ab^	22.28 ^b^	19.85 ^ab^	21.02
SeI	20.41 ^ab^	20.94 ^ab^	20.53 ^ab^	20.63
SeII	20.26 ^ab^	20.32 ^ab^	19.13 ^a^	19.90
Average	20.53	21.18	19.84	20.52
2nd selenium application date (C)				
Se0	20.94 ^ab^	22.28 ^b^	19.85 ^ab^	21.02
SeI	19.69 ^ab^	20.32 ^ab^	21.00 ^ab^	20.34
SeII	21.16 ^ab^	20.35 ^ab^	19.29 ^ab^	20.26
Average	20.60	20.98	20.05	20.54
Average				
Se0	20.94	22.28	19.85	21.02
SeI	20.05	20.63	20.77	20.49
SeII	20.71	20.34	19.21	20.08
Average	20.57	21.08	19.95	20.53
LSD_0.05_	A—n.s., B—0.85, C—n.s., A × B—1.95, A × C—n.s., B × C—n.s., A × B × C—n.s.			
Common wheat (*Triticum aestivum* L.)				
1st selenium application date (C)				
Se0	20.22 ^a^	20.16 ^a^	20.10 ^a^	20.16
SeI	19.94 ^a^	20.29 ^a^	20.41 ^a^	20.21
SeII	18.88 ^a^	20.13 ^a^	19.26 ^a^	19.42
Average	19.68	20.19	19.92	19.93
2nd selenium application date (C)				
Se0	20.22 ^a^	20.16 ^a^	20.10 ^a^	20.16
SeI	20.53 ^a^	20.16 ^a^	20.07 ^a^	20.25
SeII	20.41 ^a^	19.69 ^a^	20.01 ^a^	20.04
Average	20.39	20.00	20.06	20.15
Average				
Se0	20.22	20.16	20.10	20.16
SeI	20.24	20.23	20.24	20.23
SeII	19.65	19.91	19.64	19.73
Average	20.04	20.10	19.99	20.04
LSD_0.05_	A—n.s., B—n.s., C—n.s., A × B—n.s., A × C—n.s., B × C—n.s., A × B × C—n.s.			

Values marked with the same letters do not differ significantly (*p* ≤ 0.05). LSD for: A—sulphur dose, B—selenium dose, C—selenium application date, A × B, A × C, B × C, A × B × C—interactions; significant differences at *p* ≤ 0.05; n.s.—differences not significant.

**Table 2:** Total nitrogen content (g kg^−1^ d.m.) of straw of spelt wheat (*Triticum spelta* L.) and common wheat (*Triticum aestivum* L.).

Selenium Dose (B)	Sulphur Dose (A)			Average
	S0	SI	SII	
Spelt wheat (*Triticum spelta* L.)				
1st selenium application date (C)				
Se0	5.66 ^a^	4.98 ^a^	5.78 ^a^	5.47
SeI	5.57 ^a^	5.58 ^a^	5.60 ^a^	5.72
SeII	5.04 ^a^	5.10 ^a^	4.67 ^ab^	5.00
Average	5.42	5.22	5.35	5.40
2nd selenium application date (C)				
Se0	5.66 ^a^	4.76	5.78 ^a^	5.47
SeI	5.10 ^a^	5.11	5.13 ^a^	5.27
SeII	5.20 ^a^	5.46	4.95 ^a^	5.25
Average	5.35	5.11	5.29	5.34
Average				
Se0	5.66	4.98	5.78	5.47
SeI	5.34	5.58	5.57	5.50
SeII	5.12	5.35	4.90	5.13
Average	5.39	5.30	5.42	5.37
LSD_0.05_	A—n.s., B—n.s., C—n.s., A × B—n.s., A × C—n.s., B × C—n.s., A × B × C—n.s.			
Common wheat (*Triticum aestivum* L.)				
1st selenium application date (C)				
Se0	4.61 ^a^	5.07 ^a^	4.79 ^a^	4.82
SeI	5.16 ^a^	4.67 ^a^	5.23 ^a^	5.02
SeII	5.60 ^a^	5.44 ^a^	4.79 ^a^	5.28
Average	5.12	5.06	4.94	5.04
2nd selenium application date (C)				
Se0	4.61 ^a^	5.07 ^a^	4.79 ^a^	4.82
SeI	5.32 ^a^	5.17 ^a^	5.44 ^a^	5.31
SeII	5.61 ^a^	5.95 ^a^	5.32 ^a^	5.63
Average	5.18	5.39	5.18	5.25
Average				
Se0	4.61	5.07	4.79	4.82
SeI	5.24	4.92	5.34	5.17
SeII	5.29	5.70	5.06	5.46
Average	5.05	5.23	5.06	5.15
LSD_0.05_	A—n.s., B—n.s., C—n.s., A × B—n.s., A × C—n.s., B × C—n.s., A × B × C—n.s.			

Values marked with the same letters do not differ significantly (*p* ≤ 0.05). LSD for: A—sulphur dose, B—selenium dose, C—selenium application date, A × B, A × C, B × C, A × B × C—interactions; significant differences at *p* ≤ 0.05; n.s.—differences not significant.

**Table 3:** Protein nitrogen content (g kg^−1^ d.m.) of grain of spelt wheat (*Triticum spelta* L.) and common wheat (*Triticum aestivum* L.).

Selenium Dose (B)	Sulphur Dose (A)			Average
	S0	SI	SII	
Spelt wheat (*Triticum spelta* L.)				
1st selenium application date (C)				
Se0	17.00 ^abc^	19.31 ^c^	18.04 ^bc^	18.11
SeI	18.05 ^bc^	17.15 ^abc^	18.71 ^bc^	17.97
SeII	17.12 ^bc^	17.66 ^bc^	14.94 ^a^	16.57
Average	17.39 ^abc^	18.04	17.23	17.55
2nd selenium application date (C)				
Se0	17.00 ^abc^	19.31 ^c^	18.04 ^bc^	18.11
SeI	17.19 ^abc^	16.80 ^abc^	16.88 ^abc^	16.96
SeII	18.05 ^bc^	16.22 ^ab^	16.91 ^abc^	17.06
Average	17.41	17.44	17.27	17.38
Average				
Se0	17.00	19.31	18.04	18.11
SeI	17.62	16.98	17.80	17.47
SeII	17.59	16.94	15.93	16.82
Average	17.40	17.74	17.26	17.47
LSD_0.05_	A—n.s., B—0.72, C—n.s., A × B—1.66, A × C—n.s., B × C—n.s., A × B × C—2.63			
Common wheat (*Triticum aestivum* L.)				
1st selenium application date (C)				
Se0	17.32 ^abcd^	19.00 ^d^	16.07 ^ab^	17.46
SeI	16.61 ^abc^	16.94 ^abcd^	17.83 ^abcd^	17.13
SeII	17.64 ^abcd^	16.05 ^ab^	15.31 ^a^	16.34
Average	17.19	17.33	16.40	16.99
2nd selenium application date (C)				
Se0	17.32 ^abcd^	19.00 ^d^	16.07 ^ab^	17.46
SeI	18.19 ^abcd^	17.15 ^abcd^	18.07 ^abcd^	17.80
SeII	19.26 ^cd^	18.36 ^bcd^	17.29 ^abcd^	18.31
Average	18.26	18.17	17.14	17.86
Average				
Se0	17.32	19.00	16.07	17.46
SeI	17.40	17.05	17.95	17.47
SeII	18.52	17.21	16.30	17.35
Average	17.73	17.91	16.77	17.48
LSD_0.05_	A—0.78, B—n.s., C—0.53, A × B—1.78, A × C—n.s., B × C—1.34, A × B × C—n.s.			

Values marked with the same letters do not differ significantly (*p* ≤ 0.05). LSD for: A—sulphur dose, B—selenium dose, C—selenium application date, A × B, A × C, B × C, A × B × C—interactions; significant differences at *p* ≤ 0.05; n.s.—differences not significant.

**Table 4:** Protein nitrogen content (g kg^−1^ d.m.) of straw of spelt wheat (*Triticum spelta* L.) and common wheat (*Triticum aestivum* L.).

Selenium Dose (B)	Sulphur Dose (A)			Average
	S0	SI	SII	
Spelt wheat (*Triticum spelta* L.)				
1st selenium application date (C)				
Se0	4.68 ^ab^	4.01 ^a^	4.61 ^ab^	4.44
SeI	5.01 ^ab^	4.31 ^a^	5.04 ^b^	4.80
SeII	4.50 ^ab^	4.14 ^a^	4.23 ^a^	4.29
Average	4.73	4.16	4.63	4.51
2nd selenium application date (C)				
Se0	4.68 ^ab^	4.01 ^a^	4.61 ^ab^	4.44
SeI	4.43 ^ab^	4.35 ^a^	5.66 ^a^	4.32
SeII	4.39 ^ab^	4.78 ^ab^	4.67 ^a^	4.44
Average	4.50	4.38	4.96	4.40
Average				
Se0	4.68	4.01	4.61	4.44
SeI	4.72	4.35	4.62	4.56
SeII	4.44	4.46	4.15	4.36
Average	4.61	4.27	4.46	4.45
LSD_0.05_	A—n.s., B—0.33, C—n.s., A × B—0.75, A × C—0.57, B × C—n.s., A × B × C—n.s.			
Common wheat (*Triticum aestivum* L.)				
1st selenium application date (C)				
Se0	3.48 ^a^	4.12 ^ab^	3.36 ^a^	3.66
SeI	4.20 ^ab^	3.84 ^ab^	3.92 ^ab^	3.99
SeII	4.44 ^ab^	4.18 ^ab^	3.87 ^ab^	4.16
Average	4.04	4.05	3.72	3.94
2nd selenium application date (C)				
Se0	3.48 ^a^	4.12 ^ab^	3.36 ^a^	3.66
SeI	4.25 ^ab^	4.30 ^ab^	3.61 ^ab^	4.05
SeII	4.29 ^ab^	4.84 ^b^	4.29 ^ab^	4.47
Average	4.01	4.42	3.76	4.06
Average				
Se0	3.48	4.12	3.36	3.66
SeI	4.22	4.07	3.77	4.02
SeII	4.43	4.51	4.08	4.32
Average	4.03	4.24	3.74	4.00
LSD_0.05_	A—0.35, B—0.35, C—n.s., A × B—n.s., A × C—n.s., B × C—n.s., A × B × C—n.s.			

Values marked with the same letters do not differ significantly (*p* ≤ 0.05). LSD for: A—sulphur dose, B—selenium dose, C—selenium application date, A × B, A × C, B × C, A × B × C—interactions; significant differences at *p* ≤ 0.05; n.s.—differences not significant.

**Table 5:** Ammonium nitrogen content (mg kg^−1^ d.m.) of grain of spelt wheat (*Triticum spelta* L.) and common wheat (*Triticum aestivum* L.).

Selenium Dose (B)	Sulphur Dose (A)			Average
	S0	SI	SII	
Spelt wheat (*Triticum spelta* L.)				
1st selenium application date (C)				
Se0	270.08 ^abc^	314.16 ^abc^	328.50 ^abc^	304.25
SeI	320.02 ^abc^	253.16 ^abc^	222.19 ^ab^	265.12
SeII	326.72 ^c^	237.84 ^abc^	372.85 ^ab^	312.47
Average	305.61	268.39	307.84	293.95
2nd selenium application date (C)				
Se0	270.08 ^abc^	314.16 ^abc^	328.50 ^abc^	304.25
SeI	213.31 ^ab^	236.73 ^abc^	229.71 ^ab^	226.59
SeII	201.53 ^a^	353.29 ^bc^	328.40 ^abc^	294.41
Average	228.31	301.39	295.54	275.08
Average				
Se0	270.08	314.16	328.50	304.25
SeI	266.67	244.95	225.95	245.86
SeII	264.13	295.57	350.63	303.44
Average	266.96	284.89	301.69	284.52
LSD_0.05_	A—n.s., B—36.04, C—n.s., A × B—82.80, A × C—62.06, B × C—n.s., A × B × C—131.50			
Common wheat (*Triticum aestivum* L.)				
1st selenium application date (C)				
Se0	327.83 ^abc^	369.57 ^bc^	280.85 ^ab^	326.08
SeI	343.79 ^abc^	363.88 ^abc^	361.58 ^abc^	356.42
SeII	308.19 ^ab^	389.11 ^c^	294.14 ^abc^	330.48
Average	326.60	374.19	312.19	337.66
2nd selenium application date (C)				
Se0	327.83 ^abc^	369.57 ^bc^	280.85 ^ab^	326.08
SeI	288.35 ^a^	282.50 ^ab^	296.64 ^abc^	289.16
SeII	307.78 ^abc^	323.48 ^abc^	373.86 ^bc^	335.04
Average	307.99	325.18	317.11	316.76
Average				
Se0	327.83	369.57	280.85	326.08
SeI	316.07	323.19	329.11	322.79
SeII	307.99	356.30	334.00	332.76
Average	317.30	349.69	314.65	327.21
LSD_0.05_	A—28.79, B—n.s., C—n.s., A × B—66.14, A × C—n.s., B × C—49.57, A × B × C—n.s.			

Values marked with the same letters do not differ significantly (*p* ≤ 0.05). LSD for: A—sulphur dose, B—selenium dose, C—selenium application date, A × B, A × C, B × C, A × B × C—interactions; significant differences at *p* ≤ 0.05; n.s.—differences not significant.

**Table 6:** Ammonium nitrogen content (mg kg^−1^ d.m.) of straw of spelt wheat (*Triticum spelta* L.) and common wheat (*Triticum aestivum* L.).

Selenium Dose (B)	Sulphur Dose (A)			Average
	S0	SI	SII	
Spelt wheat (*Triticum spelta* L.)				
1st selenium application date (C)				
Se0	215.03 ^a^	238.24 ^a^	233.08 ^a^	228.79
SeI	292.71 ^a^	285.58 ^a^	260.79 ^a^	279.69
SeII	293.73 ^a^	370.53 ^a^	274.51 ^a^	312.93
Average	267.16	298.12	256.13	273.80
2nd selenium application date (C)				
Se0	215.03 ^a^	238.24 ^a^	233.08 ^a^	228.79
SeI	257.83 ^a^	238.59 ^a^	340.36 ^a^	278.93
SeII	275.37 ^a^	265.47 ^a^	259.77 ^a^	266.87
Average	249.41	247.43	277.74	258.19
Average				
Se0	215.03	238.24	233.08	228.79
SeI	275.27	262.09	300.58	279.31
SeII	284.55	318.00	267.14	289.90
Average	258.29	272.78	266.94	266.00
LSD_0.05_	A—n.s., B—44.45, C—n.s., A × B—n.s., A × C—n.s., B × C—n.s., A × B × C—n.s.			
Common wheat (*Triticum aestivum* L.)				
1st selenium application date (C)				
Se0	145.48 ^a^	193.68 ^abc^	163.01 ^ab^	167.39
SeI	199.76 ^bc^	197.87 ^abc^	159.68 ^ab^	185.77
SeII	202.25 ^bc^	222.47 ^c^	196.33 ^abc^	207.01
Average	182.50	204.67	173.00	186.73
2nd selenium application date (C)				
Se0	145.48 ^a^	193.68 ^abc^	163.01 ^ab^	167.39
SeI	172.02 ^abc^	207.15 ^bc^	202.83 ^bc^	194.00
SeII	191.08 ^abc^	212.18 ^bc^	224.59 ^c^	209.28
Average	169.53	204.33	196.81	190.22
Average				
Se0	145.48	193.68	163.01	167.39
SeI	185.89	202.51	181.26	189.89
SeII	196.67	217.33	210.46	208.15
Average	176.02	204.50	184.91	188.48
LSD_0.05_	A—14.61, B—14.61, C—n.s., A × B—n.s., A × C—25.15, B × C—n.s., A × B × C—n.s.			

Values marked with the same letters do not differ significantly (*p* ≤ 0.05). LSD for: A—sulphur dose, B—selenium dose, C—selenium application date, A × B, A × C, B × C, A × B × C—interactions; significant differences at *p* ≤ 0.05; n.s.—differences not significant.

**Table 7:** Nitrate nitrogen content (mg kg^−1^ d.m.) of grain of spelt wheat (*Triticum spelta* L.) and common wheat (*Triticum aestivum* L.).

Selenium Dose (B)	Sulphur Dose (A)			Average
	S0	SI	SII	
Spelt wheat (*Triticum spelta* L.)				
1st selenium application date (C)				
Se0	96.47 ^bc^	105.06 ^c^	100.63 ^c^	100.72
SeI	83.10 ^abc^	94.65 ^bc^	93.07 ^bc^	90.27
SeII	59.45 ^ab^	96.14 ^bc^	87.41 ^abc^	81.00
Average	79.67	98.62	93.70	90.66
2nd selenium application date (C)				
Se0	96.47 ^bc^	105.06 ^c^	100.63 ^c^	100.72
SeI	100.85 ^bc^	76.91 ^abc^	99.71 ^c^	92.49
SeII	78.99 ^abc^	80.78 ^abc^	54.12 ^c^	71.30
Average	92.10	87.58	84.82	88.17
Average				
Se0	96.47	105.06	100.63	100.72
SeI	91.98	85.78	96.39	91.38
SeII	69.22	88.46	70.77	76.15
Average	85.89	93.10	89.26	89.42
LSD_0.05_	A—n.s., B—10.52, C—n.s., A × B—n.s., A × C—18.11, B × C—n.s., A × B × C—n.s.			
Common wheat (*Triticum aestivum* L.)				
1st selenium application date (C)				
Se0	80.11 ^a^	105.46 ^a^	70.04 ^a^	85.20
SeI	78.45 ^a^	105.60 ^a^	94.73 ^a^	92.92
SeII	70.54 ^a^	97.49 ^a^	82.87 ^a^	83.64
Average	76.36	102.85	82.55	87.25
2nd selenium application date (C)				
Se0	80.11 ^a^	105.46 ^a^	70.04 ^a^	85.20
SeI	66.93 ^a^	70.68 ^a^	67.90 ^a^	68.51
SeII	83.81 ^a^	85.82 ^a^	85.19 ^a^	84.94
Average	76.95	87.32	74.38	79.55
Average				
Se0	80.11	105.46	70.04	85.20
SeI	72.69	88.14	81.32	80.72
SeII	77.18	91.66	84.03	84.29
Average	76.66	95.09	78.47	83.40
LSD_0.05_	A—16.66, B—n.s., C—n.s., A × B—n.s., A × C—n.s., B × C—n.s., A × B × C—n.s.			

Values marked with the same letters do not differ significantly (*p* ≤ 0.05). LSD for: A—sulphur dose, B—selenium dose, C—selenium application date, A × B, A × C, B × C, A × B × C—interactions; significant differences at *p* ≤ 0.05; n.s.—differences not significant.

**Table 8:** Nitrate nitrogen content (mg kg^−1^ d.m.) of straw of spelt wheat (*Triticum spelta* L.) and common wheat (*Triticum aestivum* L.).

Selenium Dose (B)	Sulphur Dose (A)			Average
	S0	SI	SII	
Spelt wheat (*Triticum spelta* L.)				
1st selenium application date (C)				
Se0	41.56 ^a^	35.47 ^a^	39.17 ^a^	38.73
SeI	35.32 ^a^	31.41 ^a^	37.98 ^a^	34.90
SeII	18.04 ^a^	36.48 ^a^	32.76 ^a^	29.09
Average	31.64	34.45	36.64	34.24
2nd selenium application date (C)				
Se0	41.56 ^a^	35.47 ^a^	39.17 ^a^	38.73
SeI	30.56 ^a^	26.18 ^a^	31.42 ^a^	29.39
SeII	35.08 ^a^	39.69 ^a^	36.10 ^a^	36.96
Average	35.73	33.78	35.56	35.03
Average				
Se0	41.56	35.47	39.17	38.73
SeI	32.94	28.80	34.70	32.15
SeII	26.56	38.09	34.43	33.03
Average	33.69	34.12	36.10	34.64
LSD_0.05_	A—n.s., B—n.s., C—n.s., A × B—n.s., A × C—n.s., B × C—n.s., A × B × C—n.s.			
Common wheat (*Triticum aestivum* L.)				
1st selenium application date (C)				
Se0	14.96 ^a^	24.57 ^abc^	19.77 ^abc^	19.77
SeI	31.92 ^c^	19.84 ^abc^	9.64 ^a^	20.47
SeII	26.95 ^bc^	11.40 ^abc^	17.47 ^abc^	18.61
Average	24.61	18.60	15.63	19.61
2nd selenium application date (C)				
Se0	14.96 ^a^	24.57 ^abc^	19.77 ^abc^	19.77
SeI	10.18 ^ab^	13.71 ^abc^	12.84 ^abc^	12.24
SeII	9.53 ^a^	18.36 ^abc^	12.96 ^abc^	13.62
Average	11.56	18.88	15.19	15.21
Average				
Se0	14.96	24.57	19.77	19.77
SeI	21.05	16.78	11.24	16.36
SeII	18.24	14.88	15.22	16.12
Average	18.09	18.74	15.41	17.41
LSD_0.05_	A—n.s., B—n.s., C—3.78, A × B—12.71, A × C—9.53, B × C—n.s., A × B × C—n.s.			

Values marked with the same letters do not differ significantly (*p* ≤ 0.05). LSD for: A—sulphur dose, B—selenium dose, C—selenium application date, A × B, A × C, B × C, A × B × C—interactions; significant differences at *p* ≤ 0.05; n.s.—differences not significant.

**Table 9:** Percentage share of protein nitrogen (%) in total nitrogen in grain of spelt wheat (*Triticum spelta* L.) and common wheat (*Triticum aestivum* L.).

Selenium Dose (B)	Sulphur Dose (A)			Average
	S0	SI	SII	
Spelt wheat (*Triticum spelta* L.)				
1st selenium application date (C)				
Se0	81.43 ^abc^	86.77 ^bcd^	91.25 ^d^	86.48
SeI	88.93 ^cd^	82.13 ^abc^	90.50 ^d^	87.19
SeII	84.81 ^abcd^	87.17 ^bcd^	84.69 ^a^	85.56
Average	85.06	85.36	88.81	86.41
2nd selenium application date (C)				
Se0	81.43 ^abc^	86.77 ^bcd^	91.25 ^d^	86.16
SeI	87.36 ^bcd^	82.78 ^abc^	85.91 ^ab^	83.38
SeII	85.37 ^abcd^	79.76 ^ab^	83.98 ^bcd^	84.21
Average	84.72	83.10	87.05	84.58
Average				
Se0	81.43	86.77	91.25	86.32
SeI	88.15	82.46	88.21	85.28
SeII	85.09	83.47	84.34	84.88
Average	84.89	84.23	87.93	85.50
LSD_0.05_	A—n.s., B—36.04, C—n.s., A × B—82.80, A × C—62.06, B × C—n.s., A × B × C—131.50			
Common wheat (*Triticum aestivum* L.)				
1st selenium application date (C)				
Se0	85.94 ^ab^	92.30 ^b^	80.08 ^a^	86.11
SeI	83.43 ^ab^	83.64 ^ab^	87.78 ^ab^	84.95
SeII	94.11 ^ab^	80.69 ^a^	79.96 ^a^	84.92
Average	87.83	85.54	82.61	85.33
2nd selenium application date (C)				
Se0	85.94 ^ab^	92.30 ^b^	80.08 ^a^	86.11
SeI	89.08 ^ab^	85.60 ^ab^	90.21 ^ab^	88.30
SeII	94.64 ^ab^	93.43 ^ab^	87.03 ^ab^	91.70
Average	89.89	90.44	85.77	88.70
Average				
Se0	85.94	92.30	80.08	86.11
SeI	86.26	84.62	89.00	86.62
SeII	94.38	87.06	83.50	88.31
Average	88.86	87.99	84.19	87.01
LSD_0.05_	A—28.79, B—n.s., C—n.s., A × B—66.14, A × C—n.s., B × C—49.57, A × B × C—n.s.			

Values marked with the same letters do not differ significantly (*p* ≤ 0.05). LSD for: A—sulphur dose, B—selenium dose, C—selenium application date, A × B, A × C, B × C, A × B × C—interactions; significant differences at *p* ≤ 0.05; n.s.—differences not significant.

**Table 10:** Percentage share of protein nitrogen (%) in total nitrogen in straw of spelt wheat (*Triticum spelta* L.) and common wheat (*Triticum aestivum* L.).

Selenium Dose (B)	Sulphur Dose (A)			Average
	S0	SI	SII	
Spelt wheat (*Triticum spelta* L.)				
1st selenium application date (C)				
Se0	83.33 ^a^	81.13 ^a^	80.19 ^a^	81.55
SeI	89.93 ^a^	77.89 ^a^	84.18 ^a^	84.00
SeII	89.59 ^a^	81.61 ^a^	85.20 ^a^	85.47
Average	87.62	80.21	83.19	83.67
2nd selenium application date (C)				
Se0	83.33 ^a^	81.13 ^a^	80.19 ^a^	86.16
SeI	88.13 ^a^	78.51 ^a^	82.20 ^a^	83.38
SeII	84.93 ^a^	85.04 ^a^	83.82 ^a^	84.21
Average	85.46	81.56	82.07	84.58
Average				
Se0	83.33	81.13	80.19	83.86
SeI	89.03	78.20	83.19	83.69
SeII	87.26	83.33	84.51	84.84
Average	86.54	80.89	82.63	84.13
LSD_0.05_	A—n.s., B—n.s., C—n.s., A × B—n.s., A × C—n.s., B × C—n.s., A × B × C—n.s.			
Common wheat (*Triticum aestivum* L.)				
1st selenium application date (C)				
Se0	76.39 ^a^	82.12 ^a^	70.23 ^a^	76.25
SeI	82.24 ^a^	82.99 ^a^	76.11 ^a^	80.45
SeII	81.04 ^a^	76.76 ^a^	82.36 ^a^	80.05
Average	79.89	80.62	76.23	78.92
2nd selenium application date (C)				
Se0	76.39 ^a^	82.12 ^a^	70.23 ^a^	76.25
SeI	80.52 ^a^	84.07 ^a^	67.76 ^a^	77.45
SeII	76.92 ^a^	81.90 ^a^	81.58 ^a^	80.13
Average	77.94	82.70	73.19	77.94
Average				
Se0	76.39	82.12	70.23	76.25
SeI	81.38	83.53	71.94	78.95
SeII	78.98	79.33	81.97	80.09
Average	78.92	81.66	74.71	78.43
LSD_0.05_	A—n.s., B—10.95, C—n.s., A × B—0.75, A × C—n.s., B × C—n.s., A × B × C—n.s.			

Values marked with the same letters do not differ significantly (*p* ≤ 0.05). LSD for: A—sulphur dose, B—selenium dose, C—selenium application date, A × B, A × C, B × C, A × B × C—interactions; significant differences at *p* ≤ 0.05; n.s.—differences not significant.

## Data Availability

Data are contained within the article.
